# Characterization of key transcription factors as molecular signatures of HPV‐positive and HPV‐negative oral cancers

**DOI:** 10.1002/cam4.983

**Published:** 2017-02-03

**Authors:** Gaurav Verma, Kanchan Vishnoi, Abhishek Tyagi, Mohit Jadli, Tejveer Singh, Ankit Goel, Ankita Sharma, Kiran Agarwal, Subhash Chandra Prasad, Durgatosh Pandey, Shashi Sharma, Ravi Mehrotra, Sukh Mahendra Singh, Alok Chandra Bharti

**Affiliations:** ^1^ Division of Molecular OncologyInstitute of Cytology & Preventive Oncology (ICMR)NoidaUttar PradeshIndia; ^2^ School of BiotechnologyBanaras Hindu UniversityVaranasiUttar PradeshIndia; ^3^ Molecular Oncology LaboratoryDepartment of ZoologyUniversity of DelhiDelhiIndia; ^4^ Subharti Dental CollegeMeerutUttar PradeshIndia; ^5^ Lady Harding Medical CollegeNew DelhiIndia; ^6^ Department of OncosurgeryDr. Bheem Rao Ambedkar Institute‐Rotary Cancer HospitalAll India Institute Of Medical SciencesNew DelhiIndia

**Keywords:** Head and neck cancer, HPV‐negative, HPV‐positive, immunohistochemistry, oral squamous cell carcinoma, oropharyngeal squamous cell carcinoma, transcription factors

## Abstract

Prior studies established constitutively active AP‐1, NF‐*κ*B, and STAT3 signaling in oral cancer. Differential expression/activation of specific members of these transcription factors has been documented in HPV‐positive oral lesions that respond better to therapy. We performed a comprehensive analysis of differentially expressed, transcriptionally active members of these pivotal signaling mediators to develop specific signatures of HPV‐positive and HPV‐negative oral lesions by immunohistochemical method that is applicable in low‐resource settings. We examined a total of 31 prospective and 30 formalin‐fixed, paraffin‐embedded tissues from treatment‐naïve, histopathologically and clinically confirmed cases diagnosed as oral or oropharyngeal squamous cell carcinoma (OSCC/OPSCC). Following determination of their HPV status by GP5 + /GP6 +  PCR, the sequential sections of the tissues were evaluated for expression of JunB, JunD, c‐Fos, p50, p65, STAT3, and pSTAT3(Y705), along with two key regulatory proteins pEGFR and p16 by IHC. Independent analysis of JunB and p65 showed direct correlation with HPV positivity, whereas STAT3 and pSTAT3 were inversely correlated. A combined analysis of transcription factors revealed a more restrictive combination, characterized by the presence of AP‐1 and NF‐*κ*B lacking involvement of STAT3 that strongly correlated with HPV‐positive tumors. Presence of STAT3/pSTAT3 with NF‐*κ*B irrespective of the presence or absence of AP‐1 members was present in HPV‐negative lesions. Expression of pSTAT3 strongly correlated with all the AP‐1/NF‐*κ*B members (except JunD), its upstream activator pEGFR^Y^ ^1092^, and HPV infection‐related negative regulator p16. Overall, we show a simple combination of AP‐1, NF‐*κ*B, and STAT3 members’ expression that may serve as molecular signature of HPV‐positive lesions or more broadly the tumors that show better prognosis.

## Introduction

Despite being most preventable malignancies associated with tobacco use, squamous cell carcinoma of oral cavity retains the 11th position among the most common cancers. Oral cancer alone accounted for an estimated 300,000 new cases and 145,000 deaths in 2012 with an estimated burden of 702,000 prevalent cases over a period of 5 years 1. India alone accounts for one‐fifth of global oral cancer incidence and one‐fourth of the oral cancer mortality 2. Several molecular epidemiological studies suggest that infection of human papillomaviruses (HPVs) may be etiologically involved in a subset of head and neck cancers that include cancers of the oral cavity 3. Unlike cervical cancer, where all the carcinogenic outcomes are attributed to HPV infection, assessment of HPV's relative contribution in oral cancer development is confounded by its multifactorial etiology, largely attributed to tobacco and alcohol abuse 3. Trends reported in past two decades demonstrate a rise in the incidence of oral cancers, in spite of strong anti‐tobacco drives 4. Mere presence of HPV DNA in oral cancer, however, is not adequate to prove the cancer as virus‐driven and it might possibly reflect a transient infection unrelated to the carcinogenic process 5, 6, 7. Nevertheless, HPV‐positive oral cancers appear to have a distinct set of pathological features and are clinically viewed to be distinct from HPV‐negative cancers 8. Moreover, HPV‐positive oral cancers display well‐differentiated tumors 9, and the prevalence is higher in younger age group with no prior history of tobacco and/or alcohol consumption 10, 11, 12. Further, the HPV‐positive oral cancers show a better prognosis in terms of overall and disease‐free survival compared to the HPV‐negative cancers of comparable stage 10, 11.

Interestingly, tumors with transcriptionally inactive HPV infection (negative for viral‐RNA or E6/E7 expression) behaved clinically and pathologically similar to HPV DNA—negative tumors, as they had similar gene expression profiles and showed similar 5‐year survival rates^5^, ^6^, ^13^. On the other hand, expression of E6/E7 oncogenes was associated with better prognosis. Viral transcription is largely dependent upon the availability of a specific set of host transcription factors such as Oct‐1, NFA, TEF‐2, NF‐1, AP‐1, NF‐*κ*B, and STAT3 that derive or regulate viral gene expression by interacting with the enhancers present in the URR region in vicinity of viral promoters 14, 15, 16. In this context, independent investigations carried out by our group and others in HPV infection‐associated cancers, including cervical, oral, and tongue cancers have demonstrated involvement of aberrantly expressed and constitutively activated transcription factors AP‐1^17^, ^18^, NF‐*κ*B 19, and STAT3 20, 21. We reported differential expression of key members of these pathways in oral carcinogenesis. We found specific upregulation of AP‐1 members c‐Jun, JunB, JunD, and c‐Fos 17, whereas p50 and p65 were the overexpressed members in NF‐*κ*B family that participated in DNA binding 19. Similarly, phospho‐STAT3 (Y705), an active member of STAT3 signaling was detected in oral cancer 21. NF‐*κ*B p50 was found overexpressed and activated in oral cancer; though participation of p65 was detected particularly in HPV‐positive oral cancer 19. A recent study showed a global effect on the gene expression profile of head and neck cancers due to involvement of AP‐1, NF‐*κ*B, and STAT3. Therefore, changes in expression pattern of these key transcription factors may be responsible for biologic variability in lesional behavior and varied treatment response of HPV‐positive and HPV‐negative oral cancer patients 22.

Considering HPV transmission is solely through sexual contact 8, presence of HPV in oral cavity is slightly unusual. Not all subjects get chance of exposure to HPV, particularly in head and neck region because of variability in the practice of oral sex that results in oro‐genital transmission 23, 24, 25. Thus, it is likely that HPV‐positive tumors represent only a specific set of cases, where the host cell environment and milieu of transcription factors is favorable for HPV transcription. HPV‐infected oral cancers possibly represent the subset of oral cancers which possess the distinct milieu of host cell transcription factors. Therefore, the transcription factor profile could be a direct indicator of the clinical response manifested by HPV‐positive/HPV‐negative tumors. In view of the pivotal role of AP‐1, NF‐*κ*B, and STAT3 that independently and/or in conjunction with each other control several downstream genes involved in the overall manifestation of carcinogenesis 24, 25, 26, their expression pattern is most likely to provide useful prognostic leads.

The aforementioned observations prompted us to examine oral lesions for collective expression of a set of transcriptionally relevant factors in context of the HPV‐positive status to define molecular signatures of HPV‐positive lesions. These signatures can prove useful to discriminate lesions which could show better prognosis irrespective of the HPV status.

## Materials and Methods

### Clinical specimens and study design

**Figure 1:**
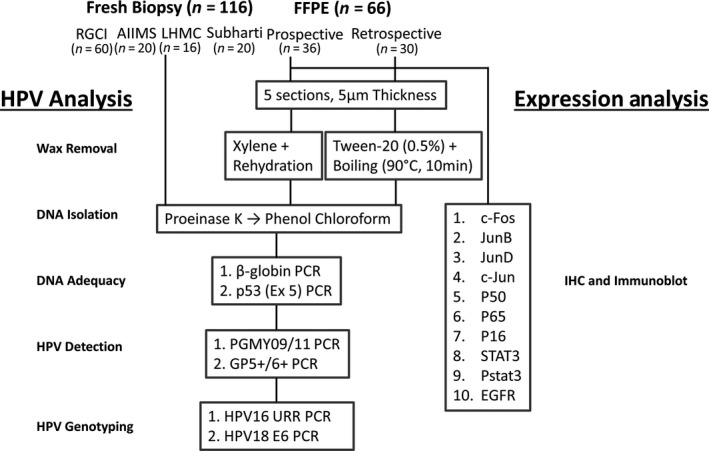
Work flow of sample collection and processing for HPV analysis and expression analysis for transcription factors. Prospectively collected, paired fresh and formalin‐fixed tissues, and retrospective tissue sections were evaluated for optimization of protocol for detection of HPV infection and expression of transcription factors in oral cancer cases by molecular methods and IHC. *Four fresh tissues and 10 paraffin blocks of noncancerous patients were also collected as controls with prior consent from dental clinic and processed similarly for HPV detection and IHC not depicted in the diagram.

A total of 116 prospectively collected fresh biopsies and 30 formalin‐fixed paraffin‐embedded preserved tissues (retrospective) from treatment‐naïve, histopathologically and clinically confirmed cases diagnosed as oral or oropharyngeal squamous cell carcinoma (OSCC/OPSCC) were obtained from three tertiary cancer care hospitals [Department of Medical Oncology, Rajiv Gandhi Cancer Institute & Research Centre (RGCI), New Delhi; Department of Surgical Oncology, All India Institute of Medical Sciences (AIIMS), New Delhi; Department of Medicine, Lady Harding Medical College (LHMC), New Delhi] and a dental hospital [Subharti Dental College and Hospital (SDCH), Meerut]. In addition, paraffin‐embedded tissue sections were also available for 36 oral cancer cases Figure 1. Four fresh tissues and 10 paraffin blocks were obtained either from normal individuals or from suspected cases but with normal histology or who attended dental clinics for unrelated problems or had undergone tooth extraction. These tissues were used as controls for the study. Prior written informed consent was obtained from all the patients and control subjects included in the study according to the principle laid down by the declaration of Helsinki, and the epidemiological details were taken from their clinical records. The clinical characteristics of these patients are presented in Table S1. DNA was isolated from fresh and paraffin sections for all the available tissues, wherein 135 cases qualified for HPV analysis. Out of 66 FFPE tissues, 61 were analyzable for HPV positivity and marker expression by IHC analysis. Fourteen fresh biopsies and four normal control tissues were available for evaluation of protein markers and other molecular investigations. All the patients were chosen prior to any chemotherapy or radiotherapy treatment. While the biopsy was sent for histopathological diagnosis in formalin solution as routine strategy, wherever possible, a portion of fresh biopsy collected in sterile cold 1× phosphate buffer saline (PBS) was immediately processed for molecular analysis or stored at −80°C until further used. The Institutional Ethics Committee of collaborating hospitals and Institute of Cytology & Preventive Oncology (ICPO), Noida, India, where the laboratory work was carried out, approved the study protocol.

### Materials

All antibodies (except *β*‐actin) and ABC staining kits were from Santa Cruz Biotechnology (Santa Cruz, CA, USA). Antibodies and their clonal description are listed in Table S2. Primers/oligonucleotides were custom‐synthesized from Microsynth (Lindau, Germany) or Eurogentec (Seraing, Belgium) listed in Table S3. Immobilon‐P PVDF membranes was from Millipore Corporation (MA, USA), xylene from Fisher Scientific (PA, USA), absolute ethanol and H_2_O_2_ were procured from Merck (CA, USA). Tween‐20, 3, 30‐Diaminobenzidine (DAB), Meyer's hematoxylin and all other reagents used in the study were of analytical and molecular biology grade and were procured from Sigma Aldrich (MO, USA) unless specified otherwise.

### Genomic DNA Isolation and HPV detection

Genomic DNA was isolated from freshly collected OSCC biopsies by the standard proteinase K digestion followed by phenol‐chloroform extraction and PCR amplification performed following the procedure described earlier 19. Genomic DNA extraction from FFPE scrape specimens (5 sections of 5 *μ*m thickness) was performed according to xylene‐free method with slight modification 27. Prior to detection of HPV infection, the adequacy of DNA was assessed by subjecting the genomic DNA (100 ng/reaction) for p53 Exon 5 PCR, which was used as internal control. HPV infection was detected by a PCR‐based method using consensus sequence‐specific primers GP5 + /GP6 +  or type‐specific primers for HPV16 and HPV18. PCR was performed in a 25 *μ*L reaction mixture containing 100 ng DNA, 10 mmol/L Tris‐HCl (pH 8.4), 50 mmol/L KCl, 1.5 mmol/L MgCl_2_, 125 mmol/L of each dNTPs (MBI fermentas, Ontario, Canada), 5pmol of oligonucleotide primers, and 0.5U Taq DNA polymerase (B. Genei (Merck), Indi) in ABI2720 Thermalcycler [Applied Biosystems Inc. (Thermo Fisher)]. The temperature profile used for amplification constituted an initial denaturation at 95°C for 4 min followed by 30 cycles of denaturation at 95°C for 30 sec, annealing at 55°C for 30 sec, and extension at 72°C for 1 min, which was extended for 5 min at the final cycle. Details of primers used for HPV detection and genotyping for HPV16 and HPV18 are presented in Table S3.

### Isolation of total protein from oral cancer tissues

Total proteins from biopsies were prepared by the method described previously 19. Briefly, the method involved a fine mincing of either fresh or frozen biopsies stored at −80°C, in chilled PBS with the help of surgical blades in a petridish kept on ice. The minced tissue material was centrifuged at 1469g at 4°C to wash off 1× PBS solution. Pellet was resuspended in lysis buffer (20 mmol/L Tris (pH 7.4), 250 mmol/L NaCl, 2 mmol/L EDTA (pH 8.0), 0.1% Triton X‐100, 0.01 mg/mL aprotinin, 0.005 mg/mL leupeptin, 0.4 mmol/L PMSF, and 4 mmol/L Na3VO4). Lysates were spun at 18001g for 10 min to remove particulate components. The concentration of proteins was then determined in the supernatant by standard Bradford assay. Protein samples were stored in small aliquots at −80°C till further use.

### Immunoblotting

Immunoblotting was performed as per routine laboratory method described earlier 19. List of specific antibodies against c‐Fos, JunB, JunD, c‐Jun, p50, p65, p16, STAT3, pSTAT3, and EGFR and dilutions are mentioned in Table S2. Detailed methodology is included as Data S3.

### Immunohistochemistry

Immunohistochemistry was performed as per routine laboratory method described earlier 19. List of specific antibodies and dilutions are mentioned in Table S2. Detailed methodology is included as Data S3.

### Histopathological and IHC evaluation

Two independent pathologists performed the histopathological evaluation of hematoxylin and eosin‐stained tissue sections as per routine procedure. Scoring of IHC staining was performed by two independent investigators (GV and AT). In cancer cases, all the cells were analyzed for c‐Fos, JunB, JunD, c‐Jun, p50, p65, p16, STAT3, pSTAT3, and EGFR immunostaining. Overall inter‐observer difference varied between 5–10%. Discrepant scores were resolved by third evaluation (ACB). Every IHC‐stained tissue was scored as reported earlier 20 on an arbitrary scale according to the number of positively stained cells and overall staining intensity of the section and assigned a value ranging from Nil (‐): no staining; Weak (+): 1–10% cells showing focal positivity; Moderate (++): 11–50% cells showing focal or diffused positivity; and Strong (+++): more than 50% cells showing diffused positivity.

### Statistical analysis

To determine association between the epidemiological and clinicopathological characteristics, Mann–Whitney test and Chi‐square test were employed, using SPSS Statistics Software version 2015 (IBM Chicago, IL, USA). Chi‐square test and Fisher's exact test (for smaller numbers on subgroup analysis) were employed to analyze association between the expression of proteins among different histopathological grades of tissue biopsies using SPSS Statistics Software version 2015 (IBM Chicago). For all analyses, *P *≤* *0.05 was considered significant.

## Results

### Identification of HPV‐positive oral cancer lesions

A total of 135 tissues out of 146 qualified for amplification of internal control for subsequent HPV analysis by GP5 + /GP6 +  PCR. Overall, 22.9% (31/135) samples were positive for HPV infection. The distribution of HPV positives was 7/30 in formalin group and 24/104 in fresh biopsies group. The clinicoepidemiological and demographic distribution of samples along with their status of HPV infection is presented in Table 1. The mean age of HPV‐positive oral cancer patients was lower than the HPV‐negative patients. An over‐representation of males was observed in oral cancer cases but HPV positivity did not differ significantly among the two genders. The samples in this study did not show any remarkable difference with respect to the habits of tobacco or alcohol abuse. Upon comparison of tumors from different subsites of the oral region, oral cavity constituted by buccal mucosa, alveolar/gingival, vestibular, retromolar space, floor of mouth, and anterior tongue showed HPV positivity of 17.6%(18/102), whereas oropharyngeal region constituted by base of tongue, tonsil, and oropharynx showed a higher HPV positivity of 39.4%(13/33; *P* = 0.038). Histologically, majority of the cases were either well‐differentiated or moderately differentiated and were in their advanced stages. HPV positivity did not display any specific distribution differences with respect to the clinical stage or histopathological grade. Analysis of type‐specific HPV infection by PCR revealed HPV16 positivity of 29% (9/31) and HPV18 positivity of 16.1% (5/31). HPV16 was detected in tumors of different subsites of oral region, whereas HPV18 was confined to buccal mucosa, alveolar/gingiva, and retro‐orbital space, or the oral cavity. HPV infection was absent in normal control tissues.

**Table 1:** HPV diagnosis and its association with different clinicoepidemiological and pathological characteristics of the oral cancer cases included in the study

Sample/case Characteristics	Overall (*n* = 135)	HPV+ve (*n* = 31)	HPV‐ve (*n* = 104)	*P* Value*
Age in Years	51.8 (±12.9)	46.2 (±13.3)	53.3 (±12.3)	**0.010** 1
Male/female ratio	110/25 (4.4:1)	27/4 (6.75:1)	83/21 (3.9:1)	
Marital status (%)				
Single	13 (9.6)	6 (19.3)	7 (6.7)	**0.038**
Married	122 (90.3)	25 (71.4)	97 (93.2)	**0.001**
Habits (%)				
Smoking only	14 (10.3)	5 (16.1)	9 (8.6)	0.230
Chewing only	19 (14.0)	5 (16.1)	14 (13.4)	0.704
Alcohol only	3 (2.2)	1 (3.2)	2 (1.9)	0.433
Smoking + chewing	13 (9.6)	5 (16.1)	8 (7.6)	0.159
Smoking + alcohol	14 (10.3)	2 (6.4)	11 (10.5)	0.496
Chewing + alcohol	3 (2.2)	2 (6.4)	1 (0.9)	0.066
All	37 (27.4)	6 (19.3)	31 (29.8)	0.252
Chewing (± Any habit)	72 (53.3)	13 (41.9)	40 (38.4)	0.726
Smoking (± Any habit)	78 (57.7)	13 (41.9)	50 (48.0)	0.551
Alcohol (± Any habit)	57 (42.2)	10 (32.2)	43 (41.3)	0.364
Tumor site (%)				
Oral cavity	**102 (75)**	**18 (58)**	**84 (80.7)**	**0.011**
Buccal mucosa	39 (28.8)	7 (22.5)	32 (30.7)	0.377
Alveolar/gingival	31 (22.9)	6 (19.3)	25 (24)	0.585
Vestibule	4 (6.5)	1 (3.2)	3 (2.8)	0.907
Retromolar space	6 (9.8)	1 (3.2)	5 (4.8)	0.704
Floor of mouth	4 (6.5)	1 (3.2)	3 (2.8)	0.907
Tongue	18 (13.3)	2 (6.4)	16 (15.3)	0.201
Oropharyngeal	**33 (25)**	**13 (41.9)**	**20 (19.2)**	**0.010**
Base of tongue	17 (12.5)	9 (29)	8 (7.6)	**0.001**
Tonsil	12 (8.8)	4 (12.9)	8 (7.6)	0.362
Oropharynx	4 (6.5)	Nil	4 (3.8)	0.272
Histopathology (%)				
WDSCC	74 (54.8)	13 (41.9)	61 (58.6)	0.103
MDSCC	50 (37.0)	14 (45.1)	36 (34.6)	0.289
PDSCC	11 (8.1)	4 (12.9)	7 (6.7)	0.269
TNM (%)				
Stage I	20 (14.8)	5 (16.1)	15 (14.4)	0.815
Stage II	5 (3.7)	Nil	5 (4.8)	0.216
Stage III	37 (27.4)	6 (19.3)	31 (29.8)	0.252
Stage IV	73 (54.0)	20 (64.5)	53 (50.9)	0.184

^1^Mann–Whitney test was used for comparison of mean age and chi‐square test was used for parametric analysis between HPV+ve and HPV‐ve populaions. *Bold value defines the statistically significant relation.

### Expression profile of AP‐1 family proteins

Expression of selected AP‐1 members, c‐Jun, JunB, JunD, and c‐Fos was evaluated. Immunoblot analysis of tissues from histopathological grade‐matched tumors from HPV‐positive and HPV‐negative cases demonstrated an increased expression of all the AP‐1 family members tested compared to control tissues. However, JunB, JunD, and c‐Fos showed a further increase in their expression in HPV‐positive oral cancers, whereas c‐Jun did not show a differential expression among HPV‐positive and HPV‐negative tissues (Fig. S1A). In situ examination of tumor tissues for expression of JunB, JunD, and c‐Fos by immunohistochemical analysis also confirmed presence of the AP‐1 members but their expression varied in HPV‐positive and HPV‐negative tissues on case to case basis (Fig. S1B). Statistical evaluation of different grades for AP‐1 members’ expression showed a strong correlation of JunB expression with HPV‐positive tumor tissues, whereas JunD and c‐Fos expression did not show any significant association with HPV positivity (Table 2).

**Table 2:** Correlation between immunohistochemical expressions of key molecular markers with HPV status of oral cancer lesions

Molecular Marker	Intensity	Normal (10) (%)	Oral Cancers		*P* Value	OR at 95% CI
			HPV+ve (16) (%)	HPV‐ve (45) (%)	Chi‐square test	
JunB	Nil/Weak	9 (90.0)	5 (31.3)	36 (80.0)	**<0.001**	8.8 (2.4‐31.81)
	Moderate/Strong	1 (10.0)	11 (68.7)	9 (20.0)		
JunD	Nil/Weak	4 (40.0)	3 (18.75)	7 (15.5)	0.767	0.79 (0.18‐3.55)
	Moderate/Strong	6 (60.0)	13 (81.25)	38 (84.4)		
c‐Fos	Nil/Weak	9 (90.0)	2 (12.5)	11 (24.4)	0.316	2.26 (0.44‐ 11.56)
	Moderate/Strong	1 (10.0)	14 (87.5)	34 (75.5)		
p50	Nil/Weak	8 (80.0)	2 (12.5)	7 (15.5)	0.767	1.28 (0.23‐6.96)
	Moderate/Strong	2 (20.0)	14 (87.5)	38 (84.4)		
p65	Nil/Weak	10 (100)	6 (37.5)	35 (77.7)	**0.003**	5.83 (1.70‐20.00)
	Moderate/Strong	0	10 (62.5)	10 (22.2)		
STAT3	Nil/Weak	10 (100)	12 (75.0)	19 (42.2)	**0.024**	0.75 (0.23‐2.41)
	Moderate/Strong	0	4 (25.0)	26 (57.7)		
pSTAT3 (Y705)	Nil/Weak	10 (100)	14 (87.5)	11 (24.4)	**<0.001**	0.04 (0.00‐0.23)
	Moderate/Strong	0	2 (12.5)	34 (75.5)		
EGFR	Nil/Weak	8 (80.0)	11 (68.7)	17 (37.7)	**0.033**	0.27 (0.08‐0.95)
	Moderate/Strong	2 (20.0)	5 (31.2)	28 (55.5)		
p16	Nil/Weak	9 (90.0)	3 (18.8)	39 (86.6)	**<0.001**	28.16 (6.15‐128.96)
	Moderate/Strong	1 (10.0)	13 (81.2)	6 (13.3)		

*P* values indicated in bold script indicate statistically significant difference in distribution ≤0.05.

### Expression profile of NF‐*κ*B family proteins

Expression of selected NF‐*κ*B members, p50 and p65 was evaluated. Immunoblot analysis of tissues from histopathological grade‐matched tumors from HPV‐positive and HPV‐negative cases demonstrated an increased expression of p50 compared to control tissues. However, p65 showed a detectable expression in HPV‐positive oral cancers (Fig. S2A). In situ examination of tumor tissues for p50 and p65 expression by immunohistochemical analysis confirmed variable presence of both the members of NF‐*κ*B family in HPV‐positive and HPV‐negative tissues (Fig. S2B). Statistical evaluation of the p50 and p65 expression with HPV positivity showed a strong correlation only between p65 and HPV infection in the tumor tissues but the frequency of overexpressed p50 was equally distributed among HPV‐positive and HPV‐negative tumors and did not show any association with HPV positivity (Table 2).

### Expression profile of STAT3 proteins

Next, we evaluated the expression of STAT3 and pSTAT3, the activated form of STAT3 phosphotyrosinated at Y705. Immunoblot analysis of HPV‐positive and HPV‐negative oral cancer tissues demonstrated an increased expression of STAT3 and pSTAT3 with reference to the control tissues. Notably, both STAT3 and pSTAT3 showed a concomitant increase in expression with increasing grades of lesions, particularly in HPV‐negative oral cancers. However, weak expression of STAT3 and pSTAT3 was observed in a small fraction of HPV‐positive lesions (Fig. S3A). In situ examination of tumor tissues confirmed varied presence of the STAT3 and pSTAT3 in oral tissues. Most of the HPV‐positive tissues demonstrated nil or weak expression (STAT3 ‐ 75%; pSTAT3 ‐ 87.5%), whereas a majority of HPV‐negative lesions expressed moderate/strong STAT3 (57.7%) or pSTAT3 (75.5%) (Fig. SB; Table 3). Statistical evaluation of different grades for STAT3 and pSTAT3 expression with HPV positivity showed an inverse correlation of STAT3 and pSTAT3 expression with HPV infection in oral cancers (Table**** 2).

**Table 3:** Scoring of oral cancer lesions based on presence of overexpressed members of transcription factors AP‐1, NF‐*κ*B, and STAT3

Scale	HPV+ve (*n* = 16)	HPV‐ve (*n* = 45)	*P* value (Fisher's Exact Test)
0	1	0	0.524
1	1	0	0.524
2	0	4	0.571
3	0	11	**0.048**
4	2	7	>0.999
5	11	18	**0.090**
6	1	5	0.997
7	0	0	–

Each member of AP‐1 (JunB, JunD, and c‐Fos), NF‐*κ*B (p50 and p65), and STAT3 (Total STAT3 and phosphorylated STAT3) were scored 0 for Nil/Weak expression and 1 for Moderate/Strong expression). Individual molecular marker scores were added and their distribution was evaluated in HPV‐positive and HPV‐negative oral cancer lesions by Fisher's Exact Test.

### Correlative assessment of differentially expressed transcription factors in HPV‐positive and HPV‐negative oral cancers

To quantitatively evaluate load of different members of the three signaling pathways, each member of AP‐1 (JunB, JunD, and c‐Fos), NF‐*κ*B (p50 and p65), and STAT3 (total STAT3 and phosphorylated STAT3) were scored 0 for nil or weak expression and 1 for moderate or strong expression. Individual molecular marker scores were combined and their distribution was evaluated in HPV‐positive and negative oral cancer lesions by Fisher's test (Table 3) and Box‐plot analysis (Fig. S4). HPV‐positive oral cancers demonstrated a more restrictive spread with a typical transcription factor score ranging from 4 to 5. On the other hand, negative oral lesions varied significantly with respect to their transcription factor score. Further, coexpression analysis was performed on members of AP‐1, NF‐кB, and STAT3 family of transcription factors to examine their interactions during HPV infection in oral cancer lesions. Each member of AP‐1 (JunB, JunD, and c‐Fos), NF‐*κ*B (p50 and p65), and STAT3 (total STAT3 and phosphorylated STAT3) were scored 0 for nil or weak expression and 1 for moderate or strong expression. Any marker in category AP‐1/NF‐*κ*B/STAT3 if scored as 1 in immunohistochemical analysis, indicated involvement of the respective category of the transcription factor. Based on the representation of each class (AP‐1/NF‐*κ*B/STAT3) of transcription factors, individual tumor tissues were segregated in different categories and their distribution was evaluated by paired *t*‐test. HPV‐positive oral lesions demonstrated a specific presence of AP‐1 in combination with NF‐*κ*B but with an absence of STAT3 (Table 4). The presence of STAT3 (total or the transcriptionally active pSTAT3) negatively correlated with HPV positivity even in the presence of AP‐1. None of the tissues expressed independent NF‐*κ*B or STAT3, whereas their coexpression in the absence of AP‐1 was exclusively associated with HPV‐negative tumors. Tumor tissues showing exclusive expression of AP‐1 members alone did not show any specific association with HPV positivity.

**Table 4:** Coexpression analysis of AP‐1, NF‐кB, and STAT3 members in oral cancer lesions and their association with the HPV status

Category	HPV+ve (*n* = 16)	HPV‐ve (*n* = 45)	*P* value
AP1^−^ NF‐кB^−^ STAT3^–^	1	0	0.524
AP1^+^ NF‐кB^−^ STAT3^–^	1	5	0.997
AP1^−^ NF‐кB^+^ STAT3^–^	0	0	0
AP1^−^ NF‐кB^−^ STAT3^+^	0	0	0
AP1^+^ NF‐кB^+^ STAT3^–^	**10**	**5**	**0.0002** **
AP1^+^ NF‐кB^−^ STAT3^+^	0	0	0
AP1^−^ NF‐кB^+^ STAT3^+^	0	4	0.571
AP1^+^ NF‐кB^+^ STAT3^+^	**4**	**31**	**0.005** **

Each member of AP‐1 (JunB, JunD, and c‐Fos), NF‐*κ*B (p50 and p65), and STAT3 (Total STAT3 and phosphorylated STAT3) were scored 0 for Nil/Weak expression and 1 for Moderate/Strong expression). Any marker in category AP‐1/NF‐*κ*B/STAT3 if scored as 1 in immunohistochemical analysis, indicated involvement of respective category of the transcription factor. Based on the presence or absence of each class (AP‐1/NF‐*κ*B/STAT3) of transcription factor, individual tumor tissues were segregated in different categories and their distribution was evaluated by Fisher's Exact Test. **Highly significant correlation.

### Influence of constitutively active upstream STAT3 signaling on other transcription factors

The role of STAT3 in HPV‐positive and negative oral lesions was evaluated further by examining level of pEGFR (Y1092) that activates STAT3 28 and p16, a negative regulator of STAT3 29 and an established surrogate marker for HPV infection 7. Immunoblot analysis of tissues from histopathological grade‐matched tumors from HPV‐positive and HPV‐negative oral cancer cases showed an increased expression of pEGFR and p16 with reference to control tissues. Expression of pEGFR was largely undetectable in HPV‐positive tumors using immunoblotting, whereas, HPV‐negative tumors showed marked variability in pEGFR expression (Fig. S5A). HPV‐positive tissues consistently showed strong p16 expression. p16 was also detectable in well‐differentiated or moderately differentiated lesions. p16 expression strongly correlated with HPV positivity (*P* < 0.001; OR‐28.16) (Table 2). In situ examination of tumor tissues by IHC analysis demonstrated active EGFR (pEGFR) expression in both HPV‐positive and negative tumors with higher pEGFR positivity associated with HPV‐negative tumors (*P* = 0.033) and it negatively correlated with HPV infection status (OR – 0.27) (Fig. S5B; Table 2). Next, overall relationship between STAT3/pSTAT3 expression, positive (pEGFR) and negative regulators (p16) of STAT3 signaling, and individual AP‐1 and NF‐*κ*B members in the oral cancer lesions was examined (Table 5). Interestingly, except with JunB, pSTAT3 positivity strongly correlated with all the molecular markers tested. A strong positive correlation was observed between pEGFR and p50, whereas a negative association was detected with p16, p65, and AP‐1 member (c‐Fos and JunB).

**Table 5:** Correlation between STAT3/pSTAT3 expression, positive (pEGFR) and negative regulators (p16) of STAT3 signaling, and individual AP‐1 and NF‐кB members in oral cancer lesions

		STAT			pSTAT3			EGFR			P16			P50			P65			c‐Fos			Jun B		
	Expn	M/S (%)	N/W (%)	*P*‐value	M/S (%)	N/W (%)	*P*‐value	M/S (%)	N/W (%)	*P* ‐value	M/S (%)	N/W (%)	*P*‐value	M/S (%)	N/W (%)	*P*‐value	M/S (%)	N/W (%)	*P* ‐value	M/S (%)	N/W (%)	*P* ‐value	M/S (%)	N/W (%)	*P* ‐value
pSTAT3	M/S	27 (44.3)	3 (4.9)	**<0.001**																					
	N/W	9 (14.8)	22 (36.1)																						
EGFR	M/S	18 (29.5)	12 (19.7)	0.363	25 (41.0)	11 (18.0)	**0.004**																		
	N/W	15 (24.6)	16 (26.2)		8 (13.1)	17 (27.9)																			
p16	M/S	6 (9.8)	24 (39.3)	0.064	7 (11.5)	29 (47.5)	**0.018**	8 (13.1)	25 (41.0)	0.206															
	N/W	13 (21.3)	18 (29.5)		12 (19.7)	13 (21.3)		11 (18.0)	17 (27.9)																
p50	M/S	30 (49.9)	0 (0.0)	**<0.001**	36 (59.0)	0 (0.0)	**<0.001**	32 (52.5)	1 (1.6)	**<0.005**	19 (31.1)	0 (0.0)	**0.029**												
	N/W	22 (36.1)	9 (14.8)		16 (26.2)	9 (14.8)		20 (32.8)	8 (13.1)		33 (54.1)	9 (14.8)													
p65	M/S	6 (9.8)	24 (39.3)	**0.036**	7 (11.5)	29 (47.5)	**0.008**	7 (11.5)	26 (42.6)	**0.037**	12 (19.7)	7 (11.8)	**<0.001**	18 (29.5)	34 (55.7)	0.465									
	N/W	14 (23.0)	17 (27.9)		13 (21.3)	12 (19.7)		13 (21.3)	15 (24.6)		8 (13.1)	34 (55.7)		2 (3.3)	7 (11.5)										
c‐Fos	M/S	24 (39.3)	6 (9.8)	0.806	25 (41.0)	11 (18.0)	**0.034**	25 (41.0)	8 (13.1)	0.544	15 (24.6)	4 (6.6)	0.974	18 (29.5)	2 (3.8)	0.132	18 (29.5)	2 (3.3)	0.132						
	N/W	24 (39.3)	7 (11.5)		23 (39.3)	2 (3.3)		23 (39.3)	5 (8.2)		33 (54.1)	9 (14.8)		30 (49.2)	11 (18.0)		30 (49.2)	11 (18.0)							
JunB	M/S	10 (16.4)	20 (32.8)	0.929	7 (11.5)	29 (47.5)	**0.008**	6 (9.8)	27 (44.3)	**0.008**	10 (16.4)	9 (14.8)	**0.026**	18 (29.5)	34 (55.7)	0.465	8 (13.1)	12 (19.7)	0.402	14 (23.0)	34 (55.7)	0.247			
	N/W	10 (16.4)	21 (34.4)		13 (21.3)	12 (19.7)		14 (23.0)	14 (23.0)		10 (16.4)	32 (52.5)		2 (3.3)	7 (11.5)		12 (19.7)	29 (47.5)		6 (9.8)	7 (11.5)				
JunD	M/S	25 (41.0)	5 (8.2)	0.955	28 (45.9)	8 (13.1)	0.14	28 (45.9)	5 (8.2)	0.776	14 (23.0)	5 (8.2)	0.159	44 (72.1)	8 (13.1)	0.609	17 (27.9)	3 (4.9)	0.837	44 (72.1)	4 (6.6)	**<0.001**	18 (29.5)	2 (3.3)	0.346
	N/W	26 (42.6)	5 (8.2)		23 (37.7)	2 (3.3)		23 (39.3)	5 (8.2)		37 (60.7)	5 (8.2)		7 (11.5)	2 (3.8)		34 (55.7)	7 (11.5)		7 (11.5)	6 (9.8)		33 (54.1)	8 (13.1)	

Cross‐tab analysis was carried out between each pair of molecular markers by chi‐square test. For statistical analysis, frequency of tissues with nil or weak (N/W) expression of respective marker were grouped together and compared against the frequency of tissues with moderate or strong (M/S) expression. *P* values below 0.05 were considered significant and are indicated in bold script.

## Discussion

In this study, we attempted to establish a correlation between key members of AP‐1, NF‐*κ*B, and STAT3 family and to develop their profile as diagnostic/prognostic signatures of HPV‐positive oral cancer lesions that may show better outcome by applying routine IHC methodology. The investigation revealed a simple combination of AP‐1 (any JunB/JunD/c‐Fos) and NF‐*κ*B (p50/p65) lacking STAT3 (STAT3/pSTAT3Y705), a signature that was associated with HPV‐positive tumors. On the contrary, presence of STAT3/pSTAT3 with NF‐*κ*B lacking p65 irrespective of the presence or absence of AP‐1 members was predominantly associated with HPV‐negative lesions. Although IHC is a subjective and qualitative method, it is a routine and reliable technique in most of the pathology departments and requires minimal infrastructure that can be established in low‐resource settings with relative ease.

In the study, HPV‐positive cases were of younger age group and HPV positivity was found to be typically over‐represented in males as reported by others 8. Incidence of oral cancers, in general, is higher in males 2, 8. The reason(s) why HPV positivity correlates more with male gender in oral cancer is not clear. It is likely that male hormones in combination with key etiological factors like tobacco or alcohol, may synergistically promote carcinogenic events in oral mucosal tissues. Our attempt to capture sexual behavior of patients to evaluate oro‐genital transmission failed as most of the study subjects abstained. Moreover, a higher HPV positivity was detected in oral cancer patients who reported their marital status as “single”. However, in other studies, varied sexual habits like oral sex have been implicated in genito‐oral transmission of HPV 8. On the other hand, tobacco habits (chewing and smoking) and alcohol abuse alone or in combination showed no association with HPV positivity. Large epidemiological studies aimed to examine the association of the major etiological factors demonstrated HPV infection as an independent risk factor aside from alcohol or tobacco smoking 12.

HPV infection among different oral subsites varied considerably. This is again explainable with tissue‐specific variations in expression/activity of transcription factors. Tumors from oral cavity collectively showed lesser HPV positivity than tumors of oropharyngeal region. A higher frequency of detecting HPV in oropharyngeal region is well documented 4, 30. Although, most of the patients belonged to an advanced stage of the disease (Stage III and Stage IV), the tissues examined in this study irrespective of their HPV status primarily demonstrated well or moderately differentiated tumors. There has been conflicting reports regarding association of HPV with differentiation status in the oral/oropharyngeal lesions 9. Nevertheless, finding more differentiated lesions may be due to regional differences in the study population 19, 31. The overall analysis of epidemiological and clinicopathological features of the samples and study subjects demonstrated a representative sample distribution that relatively matched global trends in HPV‐positive and negative oral cancers.

Our analysis of full spectrum of AP‐1 member in oral carcinogenesis showed involvement of AP‐1 members c‐Jun, JunB, JunD, and c‐Fos during oral carcinogenesis 17. Examination of these proteins by immunoblotting revealed a differential expression of JunB, JunD, and c‐Fos in HPV‐positive tumors. Most of the high‐risk HPVs possess AP‐1 binding sites in their LCR. Activity of AP‐1 is indispensable for transcription of viral oncogenes 14. AP‐1 is known to control expression of genes that regulate cell cycle, cell survival, and growth and participate in carcinogenesis 32. However, heterogeneity in AP‐1 complex composition fine‐tunes the process and result in expression of an overlapping but distinct set of downstream genes. Jun family members show contrasting effects. In human squamous cell carcinoma gene knockout models, c‐Jun had tumor promoter effect, whereas presence of JunB induced premature epithelial differentiation and slowed cell growth and upregulation of p16 33. Therefore, even presence of related members of AP‐1 family in HPV‐positive and HPV‐negative tumor represents a distinct gene profile that may be responsible for better response of HPV‐positive tumors.

Aside from AP‐1, HPV‐positive oral cancers also expressed key members of NF‐*κ*B family, p50, and p65, whereas presence of p65 was differentially over‐represented in HPV‐positive oral cancers. NF‐*κ*B interacts with viral promoter via a potential NF‐*κ*B binding site 15. The activity and expression of NF‐*κ*B increase in oral cancer depending on disease severity irrespective of the HPV infection 19. An enhanced expression of the functional components of NF‐кB signaling was reported in HPV‐infected tumors that resulted in differential downstream gene profile 34. p65(RelA) is a canonical partner and a key regulatory member of active NF‐*κ*B complex. However, nuclear p65 phosphorylated at Ser 276 is found to contribute to malignant phenotype and mediate chemoresistance 35, 36, 37. NF‐*κ*B along with AP‐1 members showed a strong cooperativity 38. NF‐*κ*B constituted by p50/p65 is essentially required for c‐Fos expression 26. Specific interaction of p65 with AP‐1 member c‐Jun has been reported to governs MMP‐9 transcription while interacting with its respective promoter sequence 39. MMP‐9, strong mediator of invasion, like other procarcinogenic factors requires binding of specific members of AP‐1 and NF‐кB family. It is likely that presence of JunB and JunD that are participating in HPV transcription may out‐compete c‐Jun and dampen the aggressive behavior of HPV‐positive tumors. Presence of c‐Jun AP‐1 complex was associated with aggressive HPV‐negative tongue cancer 18. Therefore, specific combinations of AP‐1 members, as observed in this study, are not only productive for HPV transcription but may represent a cocktail that govern overall behavior of even HPV‐negative tumors.

Examination of activated STAT3 (pSTAT3Y705) showed a negative association of this markers with HPV positivity in oral lesions. The results obtained are in sharp contrast to the carcinogenic role of STAT3 in HPV infection of cervical carcinogenesis 20. Nuclear localization of STAT3 in head and neck cancers was reportedly associated with a better prognosis 40. Constitutively active STAT3 has been demonstrated to promote cell survival of oral cancer cell lines 41. Activation of STAT3 is one of the early events in tobacco‐mediated oral carcinogenesis 42. STAT3 presence is commonly reported with poor prognosis, especially in conditions when it is found associated with active NF‐*κ*B 21, 37. A molecular cross‐talk between STAT3 and NF‐кB signal pathways has been reported 43 that promotes development and progression of cancer 44 and this possibly extends to AP‐1 also. IKKa and IKKb can cooperatively activate NF‐*κ*B and EGFR/AP1 networks of signaling pathways and contribute to the malignant phenotype and the intrinsic or acquired therapeutic resistance of HNSCC 45. Similarly, junB promoter contains STAT3 binding sites 46, whereas junB can negatively regulate STAT3 via expression of its negative regulator p16 29, 33.

Correlative assessment of differentially expressed members of AP‐1, NF‐кB, and STAT3 signaling in HPV‐positive and negative tumors revealed specific differences in member profiles. HPV‐positive oral cancers demonstrated a more restricted spread. HPV‐positive oral lesions demonstrated a specific presence of AP‐1 in combination with NF‐*κ*B but with an absence of STAT3. It is hypothesized that the cocktail of the transcription factors from AP‐1, NF‐кB, and STAT3 family collectively determines expression of the components of each transcription factor that further controls distinct sets of genes involved in oral cancer progression. The specific composition of these transcription factors are not only required for active HPV transcription but will differentially control the spectrum of genes expressed in the cell. High‐throughput screens of HPV‐positive and negative head and neck cancers showed differences in the gene expression patterns, which correlated to STAT3/NF‐кB/AP‐1 signaling pathway 22.

Along with active STAT3, pEGFR1092, and p16, being positive and negative regulators of STAT3 signaling, were examined. Our data showed an negative association of pEGFR with HPV infection in oral cancer. EGFR is known to regulate HPV16 transcription via AP‐1. However, in case of oral cancer, we observed its negative correlation particularly with JunB (*P* = 0.008). High pEGFR expression was accompanied with pSTAT3 levels in HPV‐negative tumors. Interestingly, HPV infection had no impact on treatment response to EGFR blockers in head and neck cancer and these inhibitors are recommended independently of HPV status 47. EGFR‐independent constitutive activation of STAT3 in head and neck cancers can also be mediated by IL‐6 48. In contrast, p16 was found to be upregulated in oral cancers. p16 is also a surrogate marker of HPV‐positive infection 49. A series of recent reports show that p16 independently performs well as potential prognostic marker as it dampens tumor invasion 29, 50. p16 is a negative regulator of STAT3 29 and notably the presence of JunB induces p16 expression independent of HPV, which works as negative regulator of STAT3 signaling 33.

**Figure 2:**
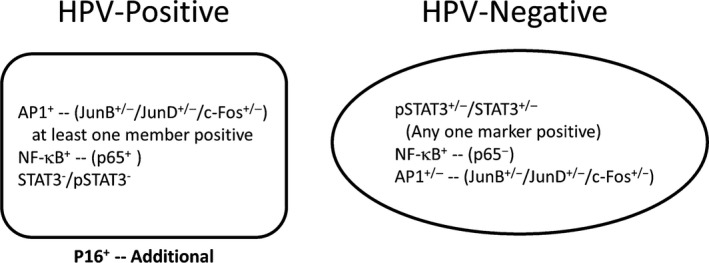
Schematic representation of functionally relevant transcription factors of AP‐1, NF‐*κ*B, and STAT3 family as molecular signatures of HPV‐positive and HPV‐negative oral cancers lesions. AP‐1 positivity is marked by expression of any of the indicated members with level classified as moderate or strong. NF‐*κ*B represented by presence of moderate or strong level of expression of p65 but absence, or if present, the expression classified not exceeding the status as low for both, STAT3 and pStat3. HPV‐negative: STAT3 and pStat3 classified as moderate or strong, NF‐*κ*B having no expression or low expression of p65 irrespective of presence or absence of any indicated family member of AP‐1.

Therefore, our study along with the available strong literature provides a useful combinatorial expression data of key members of AP‐1, NF‐*κ*B, and STAT3 that participate in DNA binding and qualify as molecular signature of HPV‐positive and negative oral cancers (Fig. 2). Expression of any member of JunB, JunD, or c‐Fos classified conventionally as moderate or strong but with absence of STAT3/pSTATY705 along with NF‐*κ*B p65 is a representative signature of HPV‐positive tumors. Expression of STAT3/pSTAT3 with absence of p65, irrespective of AP‐1 status, is a representative signature of HPV‐negative tumors. Presence of p16 served as an additional surrogate marker of HPV‐positive tumors. A large scale study is, however, required for clinical validation of these observations. Unlike p53, mutational inactivation of these transcriptional factors is rare during carcinogenesis and most of these transcription factors are regulated at expression and functional levels. The major advantage of using the set of transcription factors as a prognostic marker will be to distinguish a subset of HPV‐positive cases that may not show a good response to therapy and more importantly to identify those oral cancer lesions that did not get the chance to contract HPV but still can show better outcome.

## Conflict of Interest

The authors declare that there are no conflict of interests.

## Supporting information


**Figure S1**. Expression profile of key components of transcription factor AP‐1 family in HPV‐positive and HPV‐negative oral cancers: (A) (1) Representative immunoblots of total cellular proteins (50 *μ*g/lane) from histopathological grade‐matched HPV‐positive and HPV‐negative biopsies from oral cancer patients, tested for the expression of c‐Jun, JunB, JunD, and c‐Fos. The blots were stripped and reprobed for *β*‐actin and evaluated as input control. (2) Aggregated mean (±S.D.) abundance ratios of the band intensity of indicated proteins in HPV‐positive and HPV‐negative oral cancer tissue normalized to *β*‐actin in three independent experiments. *P ≤ 0.05 versus expression of the respective proteins in control normal tissues. #*P* ≤ 0.05 versus expression of the respective proteins in control HPV‐negative oral cancer biopsies. (B) Representative photomicrographs of immunohistochemical analysis of c‐Fos, c‐Jun, JunB, and JunD in histopathological grade‐matched HPV‐positive and HPV‐negative biopsies. Freshly fixed, paraffin‐embedded sections (5 *μ*m) of oral tissues were processed for IHC and probed for c‐Fos, c‐Jun, JunB, and JunD with respective antibodies and detected by HRP‐DAB method. Brown precipitate indicates immunopositive cells, blue stain represent nuclei, and co‐localization of brown and blue stain represents nuclear localization of respective AP‐1 family members.
**Figure S2.** Level of key components of NF‐кB family members, p50 and p65, in HPV‐positive and HPV‐negative oral cancer: (A) (1) Representative immunoblots of total cellular proteins (50 *μ*g/lane) from histopathological grade‐matched HPV‐positive and HPV‐negative biopsies from oral cancer patients, tested for the expression of p50 and p65. The blots were stripped and reprobed for *β*‐actin and evaluated as input control. (2) Aggregated mean (±SD) abundance ratios of the band intensity of indicated proteins in HPV‐positive and HPV‐negative oral cancer tissue normalized to *β*‐actin in three independent experiments. *P ≤ 0.05 versus expression of the respective proteins in control normal tissues. ^#^ *P* ≤ 0.05 versus expression of the respective proteins in control HPV‐negative oral cancer biopsies. (B) Representative photomicrographs of immunohistochemical analysis of p50 and p65 in histopathological grade‐matched HPV‐positive and HPV‐negative biopsies. Freshly fixed, paraffin‐embedded sections (5 *μ*m) of oral tissues were processed for IHC and probed for p50 and p65 with respective antibodies and detected by HRP‐DAB method. Brown precipitate indicates immunopositive cells, blue stain represent nuclei, and co‐localization of brown and blue stain represents nuclear localization of respective NF‐кB family members.
**Figure S3.** Level of key components of STAT3 and pSTAT3 in HPV‐positive and HPV‐negative oral cancer: (A). (1) Representative immunoblots of total cellular proteins (50 *μ*g/lane) from histopathological grade‐matched HPV‐positive and HPV‐negative biopsies from oral cancer patients, tested for the expression of STAT3 and pSTAT2 (Y705). The blots were stripped and reprobed for *β*‐actin and evaluated as input control. (2) Aggregated mean (±SD) abundance ratios of the band intensity of indicated proteins in HPV‐positive and HPV‐negative oral cancer tissue normalized to *β*‐actin in three independent experiments. *P ≤ 0.05 versus expression of the respective proteins in control normal tissues. ^#^ *P* ≤ 0.05 versus expression of the respective proteins in control HPV‐negative oral cancer biopsies**. (**B) Representative photomicrographs of immunohistochemical analysis of STAT3 and pSTAT2 (Y705) in histopathological grade‐matched HPV‐positive and HPV‐negative biopsies. Freshly fixed, paraffin‐embedded sections (5 *μ*m) of oral tissues were processed for IHC and probed for STAT3 and pSTAT2 (Y705) with respective antibodies and detected by HRP‐DAB method. Brown precipitate indicates immunopositive cells, blue stain represent nuclei, and co‐localization of brown and blue stain represents nuclear localization of respective STAT3.
**Figure S4.** Distribution of samples according to the individual score of expression: Each member of AP‐1 (JunB, JunD, and c‐Fos), NF‐кB (p50 and p65), and STAT3 (Total STAT3 and phosphorylated STAT3) were scored 0 for Nil/Weak expression and 1 for Moderate/Strong expression). Individual molecular marker scores were added and their distribution was evaluated in HPV‐positive and HPV‐negative oral cancer lesions by paired *t*‐test.
**Figure S5.** Expression of important regulators involved HPV‐mediated carcinogenesis in HPV‐positive and HPV‐negative oral cancer: (A) (1) Representative immunoblots of total cellular proteins (50 *μ*g/lane) from histopathological grade‐matched HPV‐positive and HPV‐negative biopsies from oral cancer patients, tested for the expression of p16 and EGFR. The blots were stripped and reprobed for *β*‐actin and evaluated as input control. (2) Aggregated mean (±SD) abundance ratios of the band intensity of indicated proteins in HPV‐positive and HPV‐negative oral cancer tissue normalized to *β*‐actin in three independent experiments. *P ≤ 0.05 versus expression of the respective proteins in control normal tissues. ^#^ *P* ≤ 0.05 versus expression of the respective proteins in control HPV‐negative oral cancer biopsies. (B) Representative photomicrographs of immunohistochemical analysis of p16 and EGFR in histopathological grade‐matched HPV‐positive and HPV‐negative biopsies. Freshly fixed, paraffin‐embedded sections (5 *μ*m) of oral tissues were processed for IHC and probed for p16 and EGFR with respective antibodies and detected by HRP‐DAB method. Brown precipitate indicates immunopositive cells, blue stain represent nuclei, and co‐localization of brown and blue stain represents nuclear localization of respective p16 and EGFR.

**Table S1.** Overall and investigation‐wise distribution of clinical specimen and respective clinicopathological characteristics of oral cancer patients.
**Table S2**. List of antibodies used in this study.
**Table S3.** List of primers used, their amplicon size, and the annealing temperatures.

**Data S1.** PPTx1.

**Data S2.** PPTx1.

**Data S3.** Material and Method.
