# Supplementary material for: Characterization of key transcription factors as molecular signatures of HPV‐positive and HPV‐negative oral cancers
Source: Cancer Med. 2017 Feb 3;6(3):591–604. doi: 10.1002/cam4.983 (PMC5345654; doi:10.1002/cam4.983)
Supplement: Supplementary file 1 — Figure S1. Expression profile of key components of transcription factor AP‐1 family in HPV‐positive and HPV‐negative oral cancers: (A) (1) Representative immunoblots of total cellular proteins (50 μg/lane) from histopathological grade‐matched HPV‐positive and HPV‐negative biopsies from oral cancer patients, tested for the expression of c‐Jun, JunB, JunD, and c‐Fos. The blots were stripped and reprobed for β‐actin and evaluated as input control. (2) Aggregated mean (±S.D.) abundance ratios of the band intensity of indicated proteins in HPV‐positive and HPV‐negative oral cancer tissue normalized to β‐actin in three independent experiments. *P ≤ 0.05 versus expression of the respective proteins in control normal tissues. #P ≤ 0.05 versus expression of the respective proteins in control HPV‐negative oral cancer biopsies. (B) Representative photomicrographs of immunohistochemical analysis of c‐Fos, c‐Jun, JunB, and JunD in histopathological grade‐matched HPV‐positive and HPV‐negative biopsies. Freshly fixed, paraffin‐embedded sections (5 μm) of oral tissues were processed for IHC and probed for c‐Fos, c‐Jun, JunB, and JunD with respective antibodies and detected by HRP‐DAB method. Brown precipitate indicates immunopositive cells, blue stain represent nuclei, and co‐localization of brown and blue stain represents nuclear localization of respective AP‐1 family members. Figure S2. Level of key components of NF‐кB family members, p50 and p65, in HPV‐positive and HPV‐negative oral cancer: (A) (1) Representative immunoblots of total cellular proteins (50 μg/lane) from histopathological grade‐matched HPV‐positive and HPV‐negative biopsies from oral cancer patients, tested for the expression of p50 and p65. The blots were stripped and reprobed for β‐actin and evaluated as input control. (2) Aggregated mean (±SD) abundance ratios of the band intensity of indicated proteins in HPV‐positive and HPV‐negative oral cancer tissue normalized to β‐actin in three independent experiments. [file CAM4-6-591-s001.docx]

**Supplementary Figure Legends**

**SF1. Expression profile of key components of transcription factor AP-1 family in HPV-positive and HPV-negative oral cancers:** **A. (i)** Representative immunoblots of total cellular proteins (50μg/lane) from histopathological grade-matched HPV-positive and HPV-negative biopsies from oral cancer patients, tested for the expression of c-Jun, JunB, JunD and c-Fos. The blots were stripped and reprobed for β-actin and evaluated as input control. **(ii)** Aggregated mean (± S.D.) abundance ratios of the band intensity of indicated proteins in HPV-positive and HPV-negative oral cancer tissue normalized to β-actin in three independent experiments. *p value ≤ 0.05 versus expression of the respective proteins in control normal tissues. #p value ≤ 0.05 versus expression of the respective proteins in control HPV-negative oral cancer biopsies. **B.** Representative photomicrographs of immunohistochemical analysis of c Fos, c Jun, JunB and JunD in histopathological grade matched HPV-positive and HPV-negative biopsies. Freshly fixed, paraffin-embedded sections (5 μm) of oral tissues were processed for IHC and probed for c Fos, c Jun, JunB and JunD with respective antibodies and detected by HRP-DAB method. Brown precipitate indicates immuno-positive cells, blue stain represent nuclei and co-localization of brown and blue stain represents nuclear localization of respective AP-1 family members (Original magnification: 100×).

**SF2. Level of key components of NF-кB family members p50 and p65 in HPV-positive and HPV-negative oral cancer**: **A. (i)** Representative immunoblots of total cellular proteins (50μg/lane) from histopathological grade matched HPV-positive and HPV-negative biopsies from oral cancer patients, tested for the expression of p50 and p65. The blots were stripped and re-probed for β-actin and evaluated as input control. **(ii)** Aggregated mean (± S.D.) abundance ratios of the band intensity of indicated proteins in HPV-positive and HPV-negative oral cancer tissue normalized to β-actin in three independent experiments. *p value ≤ 0.05 versus expression of the respective proteins in control normal tissues. #p value ≤ 0.05 versus expression of the respective proteins in control HPV-negative oral cancer biopsies. **B.** Representative photomicrographs of immunohistochemical analysis of p50 and p65 in histopathological grade matched HPV-positive and HPV-negative biopsies. Freshly fixed, paraffin-embedded sections (5 μm) of oral tissues were processed for IHC and probed for p50 and p65 with respective antibodies and detected by HRP-DAB method. Brown precipitate indicates immuno-positive cells, blue stain represent nuclei and co-localization of brown and blue stain represents nuclear localization of respective NF-кB family members (Original magnification: 100×).

**SF3. Level of key components of STAT3 and pSTAT3 in HPV-positive and HPV-negative oral cancer:** **A. (i)** Representative immunoblots of total cellular proteins (50μg/lane) from histopathological grade matched HPV-positive and HPV-negative biopsies from oral cancer patients, tested for the expression of STAT3 and pSTAT2 (Y705). The blots were stripped and re-probed for β-actin and evaluated as input control. **(ii)** Aggregated mean (± S.D.) abundance ratios of the band intensity of indicated proteins in HPV-positive and HPV-negative oral cancer tissue normalized to β-actin in three independent experiments. *p value ≤ 0.05 versus expression of the respective proteins in control normal tissues. #p value ≤ 0.05 versus expression of the respective proteins in control HPV-negative oral cancer biopsies. **B.** Representative photomicrographs of immunohistochemical analysis of STAT3 and pSTAT2 (Y705) in histopathological grade matched HPV-positive and HPV-negative biopsies. Freshly fixed, paraffin-embedded sections (5 μm) of oral tissues were processed for IHC and probed for STAT3 and pSTAT2 (Y705) with respective antibodies and detected by HRP-DAB method. Brown precipitate indicates immuno-positive cells, blue stain represent nuclei and co-localization of brown and blue stain represents nuclear localization of respective STAT3 (Original magnification: 100×).

**SF4. Distribution of samples according to the individual score of expression:** Each member of AP-1 (JunB, JunD and c-Fos), NF-кB (p50 and p65) and STAT3 (Total STAT3 and phosphorylated STAT3) were scored 0 for Nil/Weak expression and 1 for Moderate/Strong expression). Individual molecular marker scores were added and their distribution was evaluated in HPV positive and HPV-negative oral cancer lesions by paired t-test.

**SF5. Expression of important regulators involved HPV mediated carcinogenesis in HPV-positive and HPV-negative oral cancer:** **A. (i)** Representative immunoblots of total cellular proteins (50μg/lane) from histopathological grade matched HPV-positive and HPV-negative biopsies from oral cancer patients, tested for the expression of p16 and EGFR. The blots were stripped and re-probed for β-actin and evaluated as input control. **(ii)** Aggregated mean (± S.D.) abundance ratios of the band intensity of indicated proteins in HPV-positive and HPV-negative oral cancer tissue normalized to β-actin in three independent experiments. *p value ≤ 0.05 versus expression of the respective proteins in control normal tissues. #p value ≤ 0.05 versus expression of the respective proteins in control HPV-negative oral cancer biopsies. **B.** Representative photomicrographs of immunohistochemical analysis of p16 and EGFR in histopathological grade matched HPV-positive and HPV-negative biopsies. Freshly fixed, paraffin-embedded sections (5 μm) of oral tissues were processed for IHC and probed for p16 and EGFR with respective antibodies and detected by HRP-DAB method. Brown precipitate indicates immuno-positive cells, blue stain represent nuclei and co-localization of brown and blue stain represents nuclear localization of respective p16 and EGFR (Original magnification: 100×).
