# Supplementary material for: Characterization of key transcription factors as molecular signatures of HPV‐positive and HPV‐negative oral cancers
Source: Cancer Med. 2017 Feb 3;6(3):591–604. doi: 10.1002/cam4.983 (PMC5345654; doi:10.1002/cam4.983)
Supplement: Supplementary file 2 — Table S1. Overall and investigation‐wise distribution of clinical specimen and respective clinicopathological characteristics of oral cancer patients. Table S2. List of antibodies used in this study. Table S3. List of primers used, their amplicon size, and the annealing temperatures. [file CAM4-6-591-s002.docx]

**Supplementary Table 1: Overall and investigation-wise distribution of clinical specimen and respective clinico-pathological characteristics of oral cancer patients.**

| **Characteristics** | **Overall**  **(n = 146)** | **HPV Analysis**  **(n = 135)** | **Western Analysis**  **(n = 14)** | **IHC Analysis**  **(n = 61)** |
| --- | --- | --- | --- | --- |
| **Age in Years** | 51.7 (±12.6) | 51.8 (±12.9) | 54.1 (±12.0) | 48.3 (±14.3) |
| **Male/Female**  **(Ratio)** | 118/28  (4:1) | 110/25  (4.4:1) | 12/2  (6:1) | 44/17  (2.5:1) |
| **Marital Status** |  |  |  |  |
| Single | 18 (12.3%) | 13 (9.6%) | 0 | 6 (9.8%) |
| Married | 128 (87.6) | 122 (90.3%) | 14 (100%) | 55 (90.1%) |
| **Tumor Site** |  |  |  |  |
| Buccal Mucosa | 44 (30.1%) | 39 (28.8%) | 1 (7.1%) | 19 (31.1%) |
| Alveolar/Gingival | 34 (23.2%) | 31 (22.9%) | 1 (7.1%) | 16 (26.2%) |
| Vestibule | 4 (2.7%) | 4 (2.9%) | 0 | 4 (6.5%) |
| Retromolar Space | 7 (4.7%) | 6 (4.4%) | 1 (7.1%) | 4 (6.5%) |
| Floor of Mouth | 4 (2.7%) | 4 (2.9%) | 0 | 3 (4.9%) |
| Tongue | 18 (12.3%) | 18 (13.3%) | 2 (14.2%) | 6 (9.8%) |
| Base of Tongue | 17 (11.6%) | 17 (12.5%) | 3 (21.4%) | 3 (4.9%) |
| Tonsil | 12 (8.2%) | 12 (8.8%) | 6 (42.8%) | 6 (9.8%) |
| Oropharynx | 6 (4.1%) | 4 (2.9%) | 0 | 0 |
| **Histopathology** |  |  |  |  |
| WDSCC | 82 (56.1%) | 74 (54.8%) | 9 (64.2%) | 43 (70.4%) |
| MDSCC | 53 (36.3%) | 50 (37.0%) | 5 (35.7%) | 15 (24.5%) |
| PDSCC | 11 (7.5%) | 11 (8.1%) | 0 | 3 (4.9%) |
| **TNM** |  |  |  |  |
| Stage I | 20 (13.6%) | 20 (14.8%) | 0 | 3 (4.9%) |
| Stage II | 9 (6.1%) | 5 (3.7%) | 1 (7.1%) | 3 (4.9%) |
| Stage III | 40 (27.3%) | 37 (27.4%) | 6 (42.8%) | 23 (37.7%) |
| Stage IV | 77 (52.7%) | 73 (54.0%) | 7 (50.0%) | 32 (52.4%) |

**Supplementary Table ST2: List of antibodies used in the present study**

| **S. No.** | **Antibodies** | **Clonality** | **Dilution** | | **Manufacturer/Vendor** | **Catalogue No.** |
| --- | --- | --- | --- | --- | --- | --- |
|  |  |  | **WB** | **IHC** |  |  |
|  | c-Fos (H-125) | Rabbit polyclonal | 1:2000 | 1:200 | Santa Cruz | SC-7202 |
|  | JunD (329) | Rabbit polyclonal | 1:2000 | 1:200 | Santa Cruz | SC-74 |
|  | JunB (210) | Rabbit polyclonal | 1:2000 | 1:200 | Santa Cruz | SC-73 |
|  | c-Jun (N) | Rabbit polyclonal | 1:2000 | 1:200 | Santa Cruz | SC-45 |
|  | NF-kB p50 (NLS) | Goat polyclonal | 1:2000 | 1:200 | Santa Cruz | SC-114X |
|  | NF-kB p65 (A) | Rabbit polyclonal | 1:2000 | 1:200 | Santa Cruz | SC-109 |
|  | STAT3 | Mouse monoclonal | 1:2000 | 1:200 | BD Biosciences | 610190 |
|  | pSTAT3 (Y705) | Mouse monoclonal | 1:2000 | 1:200 | BD Biosciences | 612543 |
|  | HPV16/18E6 (C1P5) | Mouse monoclonal | 3:1000 | 1:200 | Santa Cruz | SC-460 |
|  | HPV16E7 (TVG710Y) | Mouse monoclonal | 3:1000 | 1:200 | Santa Cruz | SC-264 |
|  | HPV 18E7 (N-19) | Goat polyclonal | 3:1000 | 1:200 | Santa Cruz | SC-1590 |
|  | p53 (DO-1) | Mouse monoclonal | 1:500 | 1:200 | Santa Cruz | SC-126 |
|  | p16 (F-12) | Mouse monoclonal | 1:1000 | 1:200 | Santa Cruz | SC-1661 |
|  | pEGFR (Tyr10924) | Rabbit polyclonal | 1:1000 | 1:200 | Santa Cruz | SC-16802 |
|  | β Actin | Mouse monoclonal | 1:10000 |  | Sigma | A1978 |

**Supplementary Table ST3: List of primers used, their amplicon size and the annealing temperatures.**

| **Primer set** | **Target** | **Primer Sequence** | **Ampli. Size (bp)** | **Ann.**  **Temp. (ºC)** | **Reference** |
| --- | --- | --- | --- | --- | --- |
| p53 | Human P53  (Exon 5) | Forward 5’-TACTCCCCTGCCCTCAACAA-3’  Reverse 5’-CATCGCTATCTGAGCAGCGC-3’ | 184 | 61 | **(**[**Yu *et al.* 1999**](#_ENREF_6)**)** |
| GP5+/6+ | L1 HPV | Forward 5’-TTGTTACTGTGGTAGATACTAC-3'  Reverse 5’-CTTATACTAAATGTCAAATAAAAA-3’ | 127 | 55 | **(**[**Jacobs *et al.* 1995**](#_ENREF_2)**)** |
| HPV16 | URR | Forward 5’-AAGGCCAACTAAATGTCAC-3’  Reverse 5’-CTGCTTTTATACTAACCGG-3’ | 217 | 55 | **(**[**Storey *et al.* 1991**](#_ENREF_5)**)** |
| HPV18 | E6 | Forward 5’-TGAGGTACCATTGGATATTT-3’  Reverse 5’-TAGCAAAAAGCTGCTTCACGC-3’ | 100 | 55 | **(**[**Shukla *et al.* 2010**](#_ENREF_4)**)** |

Jacobs, M. V., de Roda Husman, A. M., van den Brule, A. J., Snijders, P. J., Meijer, C. J. and Walboomers, J. M. (1995). Group-specific differentiation between high- and low-risk human papillomavirus genotypes by general primer-mediated PCR and two cocktails of oligonucleotide probes. J Clin Microbiol 33(4): 901-5.

Shukla, S., Bharti, A. C., Mahata, S., Hussain, S., Hedau, S., Sharma, R., Pillai, M. R., Krishna, S., Chiplunkar, S., Tengaonkar, H. and Das, B. C. (2010). Application of a Multiplex PCR to cervical cells collected by a paper smear for the simultaneous detection of all mucosal human papillomaviruses and typing of HR HPV types 16 and 18. J Med Microbiol 59(11): 1303-10.

Storey, A., Oates, D., Banks, L., Crawford, L. and Crook, T. (1991). Anti-sense phosphorothioate oligonucleotides have both specific and non-specific effects on cells containing human papillomavirus type 16. Nucleic Acids Res 19(15): 4109-14.

Yu, F. L., Zheng, W. Y., Wang, M. Y., Bender, W., Cheerva, A. and Miller, J. (1999). The effect of 17beta-estradiol-DNA adducts on the replication of exon # 5 of the human suppressor gene p53. FEBS Lett 454(1-2): 7-10.
