# Supplementary material for: Characterization of key transcription factors as molecular signatures of HPV‐positive and HPV‐negative oral cancers
Source: Cancer Med. 2017 Feb 3;6(3):591–604. doi: 10.1002/cam4.983 (PMC5345654; doi:10.1002/cam4.983)
Supplement: Supplementary file 4 — Data S2. PPTx1. [file CAM4-6-591-s004.pptx]

## Slide 1
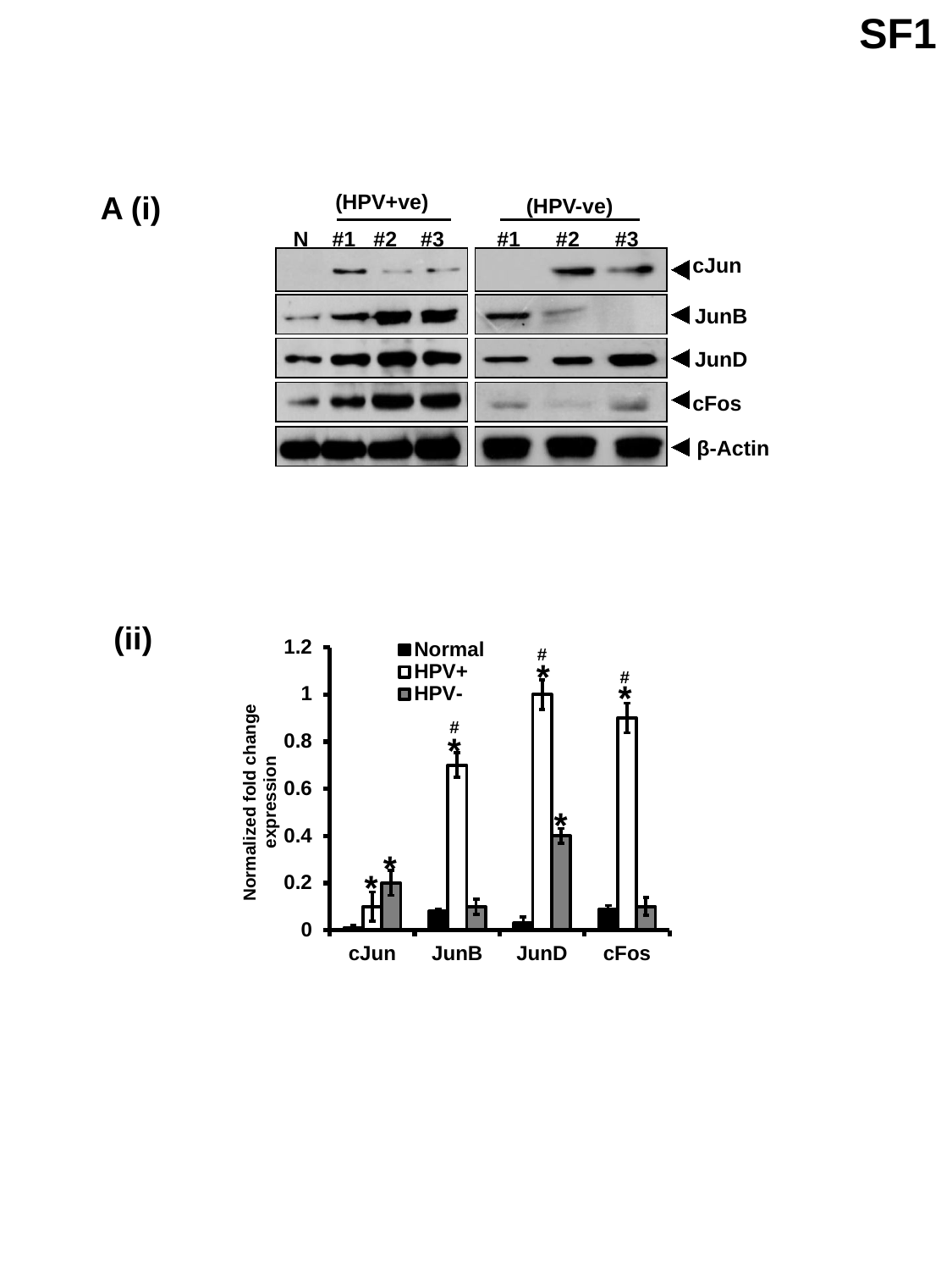

SF1
A (i)
(HPV+ve)
(HPV-ve)
N #1 #2 #3
 #1 #2 #3
cJun
JunB
JunD
cFos
β-Actin
(ii)

## Slide 2
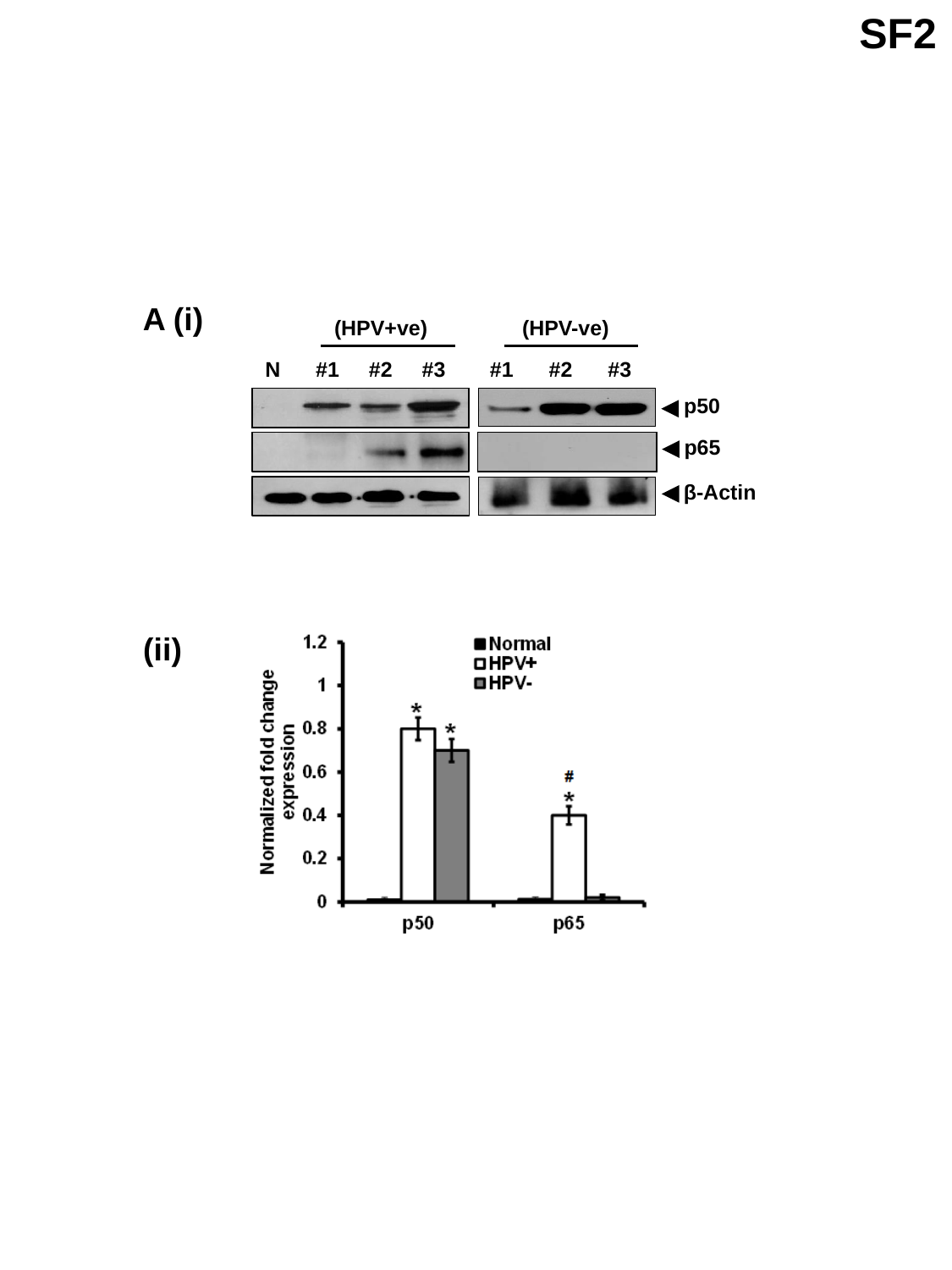

SF2
(HPV+ve)
(HPV-ve)
N #1 #2 #3
 #1 #2 #3
p50
p65
β-Actin
A (i)
(ii)

## Slide 3
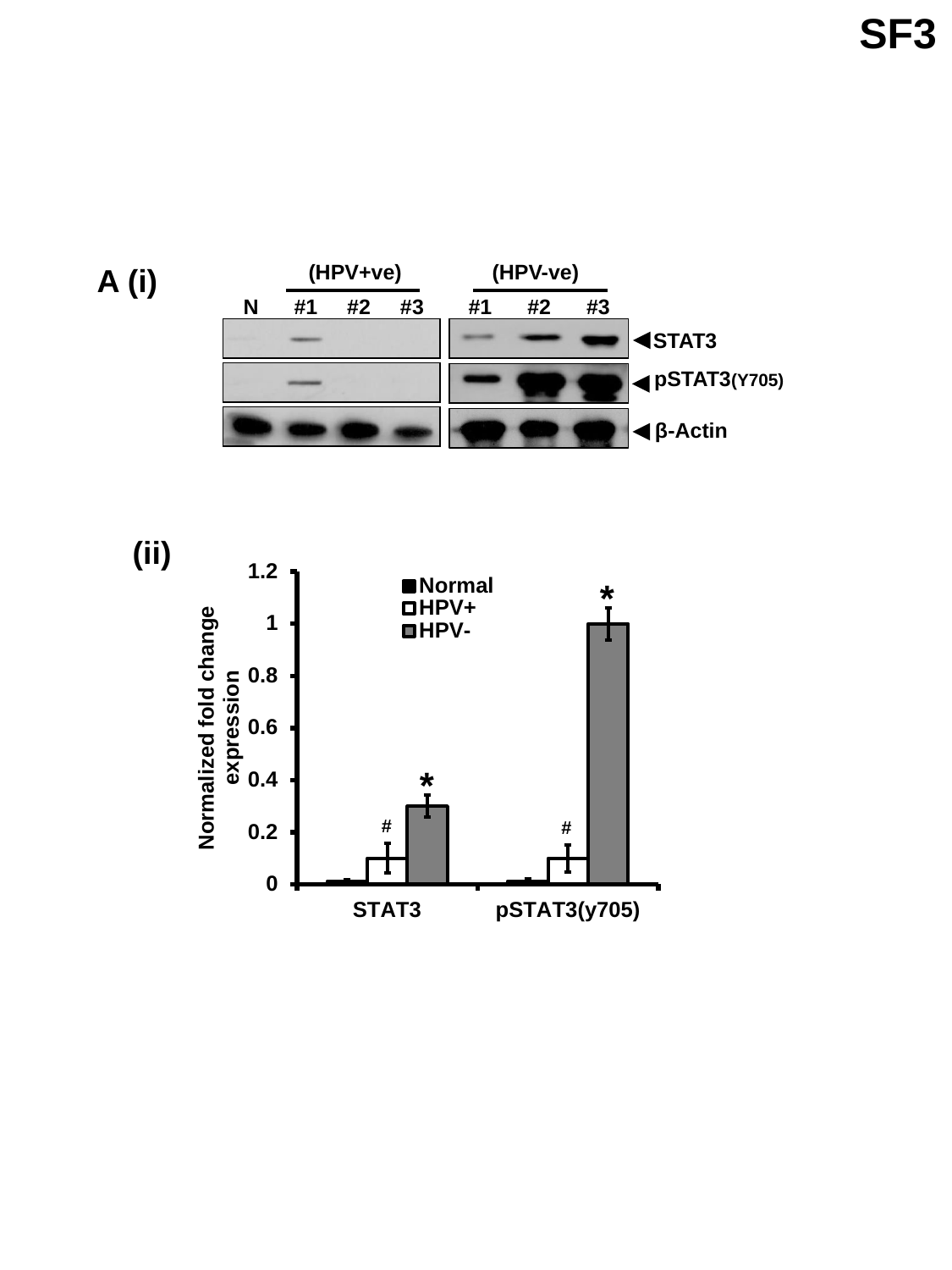

SF3
(HPV+ve)
(HPV-ve)
N #1 #2 #3
 #1 #2 #3
STAT3
pSTAT3(Y705)
β-Actin
A (i)
(ii)

## Slide 4
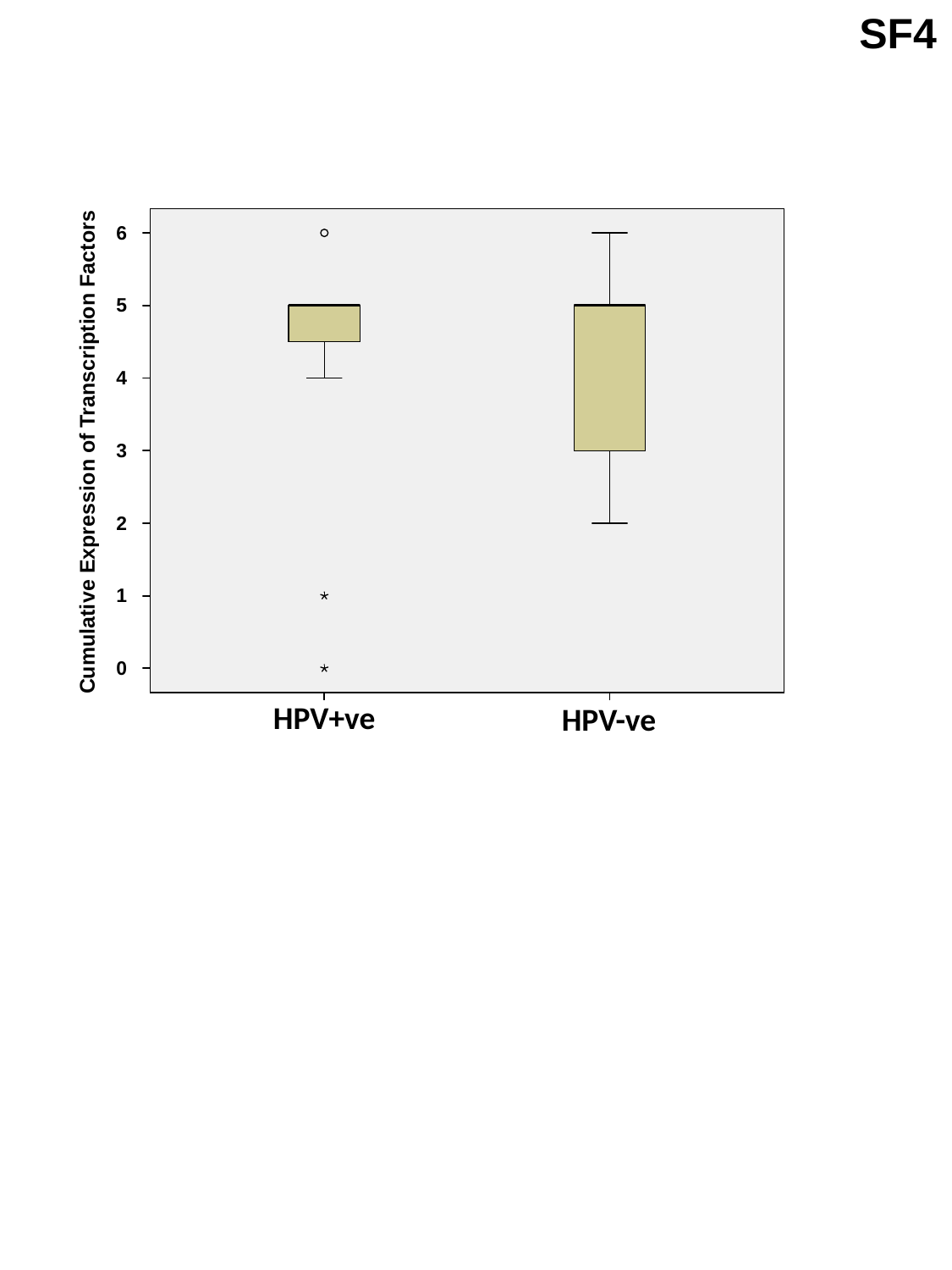

SF4
6
5
4
3
Cumulative Expression of Transcription Factors
2
1
0
HPV+ve
HPV-ve

## Slide 5
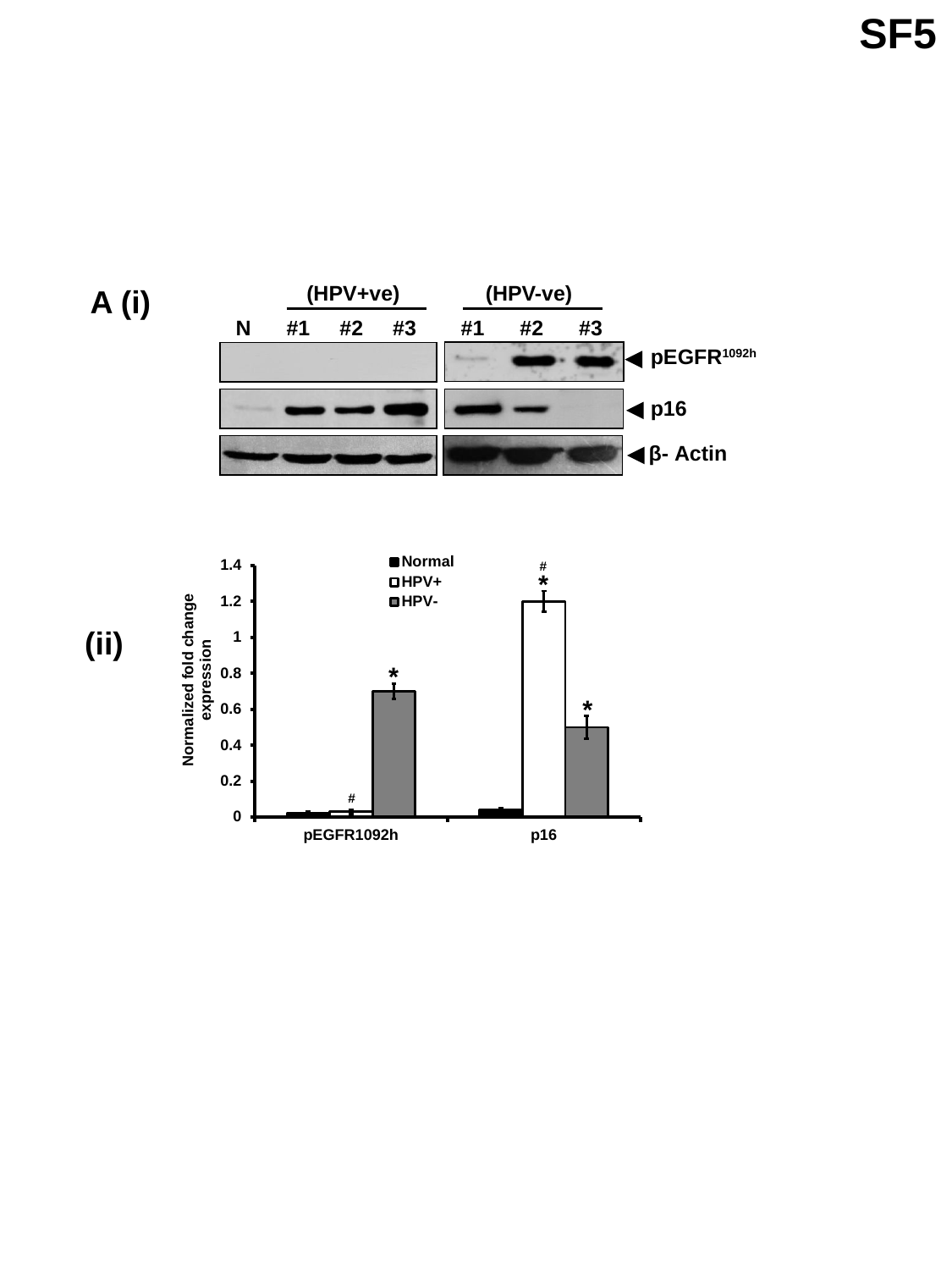

SF5
A (i)
(HPV+ve)
(HPV-ve)
N #1 #2 #3
 #1 #2 #3
pEGFR1092h
p16
β- Actin
(ii)
