# Supplementary material for: Characterization of key transcription factors as molecular signatures of HPV‐positive and HPV‐negative oral cancers
Source: Cancer Med. 2017 Feb 3;6(3):591–604. doi: 10.1002/cam4.983 (PMC5345654; doi:10.1002/cam4.983)
Supplement: Supplementary file 5 — Data S3. Material and Method. [file CAM4-6-591-s005.docx]

**Supplementary Material and Method**

**1. Immunoblotting:** Total cellular proteins (50 μg/lane) were separated in 8-12% polyacrylamide gel and electrotransferred on PVDF membranes. The membrane was blocked in PBS containing 5% non-fat skimmed milk and probed with specific antibodies against c-Fos, JunB, JunD, c-Jun, p50, p65, p16, STAT3, pSTAT3 and EGFR by incubating the membrane overnight in pre-standardized dilution of primary antibody in blocking solution at 4°C (Supplementary table ST1). These blots were washed, incubated with respective HRP-conjugated IgG secondary antibodies and visualized by Luminol detection kit and by exposing the blot to KODAK X-Omat films (Kodak India, India). The western blot membranes were reprobed for β-actin expression as an internal control. The quantitative densitometric analysis of the bands was performed using Alpha Ease FC version 4.1.0 (Alpha Innotech Corporation, IL). Expression of proteins was determined by assessing the densitometric analysis of bands visualized in immunoblot and normalized to β-actin expression.

**2.** **Immunohistochemistry:** Paraffin-embedded sections were dewaxed by washing the slides in xylene twice, each wash lasting for about 15 minutes. The sections were rehydrated first in xylene-ethanol mixture solution (xylene:ethanol; 1:1). These sections were washed in ethanol gradients (100%EtOH, 90%EtOH, 70%EtOH, 50%EtOH and 30%EtOH) for 10 minutes each. The sections were incubated with hydrogen peroxidase (3% v/v in 30% ethanol) for 30 minutes at room temperature to block the endogenous peroxidase activity. The slides were washed using Tris-Buffered Saline (TBS) (0.01M) 2 times for 10 minutes each. The antigen retrieval was done by Heat Induced Epitope Retrieval (HIER) method by heating the slides immersed in citrate buffer (10mM) in the microwave oven for 10 minutes at 800W and for 5 minutes at 400W. The slides were washed using TBS (0.01M) 2 times for 10 minutes each. Blocking was done with 1% Bovine Serum Albumin (BSA) for 1 hour at 4C in a humidified chamber, followed by washing in TBS (0.01M) 2 times for 5 minutes each. The sections on slides were incubated with a dilution of primary antibody (1:200) and kept in a humidified chamber at 4°C for 16 hours. The slides were washed using TBS (0.01M) 2 times for 10 minutes each. Excess of water was removed and the sections were incubated with secondary antibody dilution (1:300) in a moist chamber for about 1 hour at 4C. The slides were again washed using TBS (0.01M) 2 times for 10 minutes each. The avidin-biotin (1:1) peroxidase complex constituting the tertiary antibody dilution (1:300) was added to the sections appropriately and the slides were incubated for 1 hour at 4°C in a humidified chamber. The slides were washed using TBS (0.01M) 2 times for 10 minutes each. Excess of TBS was removed and the sections on slides were stained with DAB and kept still for about 1-2 minutes. The slides were washed using TBS (0.01M) 2 times for 10 minutes each. Finally, the sections were counter stained with hematoxylin for 5-10 seconds. The slides were washed first in distilled water and then in running tap water and kept for air drying. Finally, the slides were dipped in methanol and mounted using DPX and xylene and observed under the microscope.
